# A Retrospective Single-Center Cohort Study on Heme Transfusions in Orthopedic Surgeries

**DOI:** 10.7759/cureus.87028

**Published:** 2025-06-30

**Authors:** Shobhet Saxena, Hariprasad Seenappa, Subhashish Das, Nagakumar J S, Nulaka Harish, Ayush Agrawal

**Affiliations:** 1 Orthopaedics, Sri Devaraj Urs Medical College, Sri Devaraj Urs Academy of Higher Education and Research, Kolar, IND; 2 Pathology, Sri Devraj Urs Medical College, Kolar, IND; 3 Orthopaedics, Sri Devaraj Urs Academy of Higher Education and Research, Kolar, IND; 4 Orthopaedics and Traumatology, Sri Devaraj Urs Medical College, Kolar, IND; 5 Orthopaedics, Sri Devaraj Urs Medical College and RL Jalappa Hospital, Kolar, IND

**Keywords:** blood transfusion, orthopaedic surgery, patient blood management, perioperative outcomes, retrospective cohort study

## Abstract

Blood transfusions remain a critical yet potentially hazardous intervention in orthopedic surgical procedures, particularly those associated with substantial blood loss such as joint arthroplasties, spinal surgeries, and complex trauma reconstructions.

This retrospective cohort study, conducted at RL Jalappa Hospital, Kolar, India, from February 2023 to January 2024, comprehensively evaluated transfusion practices across 395 major orthopedic surgeries.

The study revealed an overall transfusion rate of 10.3%, with significant variation across procedure types, ranging from 6.8% in elective arthroplasties to 18.2% in revision surgeries and 22.4% in complex spinal procedures. Multivariate analysis identified several independent predictors of transfusion requirements, including advanced age (>65 years, OR 2.4), preoperative anemia (Hb <10 g/dL, OR 3.2), prolonged surgical duration (>3 hours, OR 2.1), and substantial intraoperative blood loss (>500 mL, OR 4.0). Importantly, transfused patients demonstrated significantly worse clinical outcomes, including extended hospital stays (7.2 vs 4.5 days, p<0.001), higher complication rates (24% vs 8%, p=0.003), particularly surgical site infections (12% vs 4%), and increased 30-day readmission rates (8% vs 3%, p=0.02). The study also evaluated institutional transfusion triggers, finding considerable variability from recommended guidelines, with 42% of transfusions administered at hemoglobin levels of >8 g/dL in hemodynamically stable patients.

These findings underscore the urgent need for standardized, evidence-based transfusion protocols, comprehensive preoperative optimization strategies, and robust blood management programs in orthopedic surgery. The study provides a framework for developing institution-specific guidelines to minimize unnecessary transfusions while optimizing patient outcomes and resource utilization.

## Introduction

Perioperative blood management in orthopedic surgery presents unique challenges due to the inherently vascular nature of musculoskeletal procedures and the demographic characteristics of typical orthopedic patients. Major orthopedic surgeries, particularly total joint arthroplasties, spinal reconstructions, and complex trauma cases, frequently involve substantial blood loss ranging from 500-2000 mL, depending on procedure complexity [1]. While allogeneic blood transfusions remain a cornerstone of management for acute blood loss anemia, growing evidence highlights their association with numerous adverse outcomes, including transfusion reactions, immunomodulation, infection transmission, and increased healthcare costs [2].

The orthopedic patient population presents particular challenges for blood management due to the high prevalence of preoperative anemia (estimated at 20-45% in elective cases), frequent use of anticoagulant medications, and the elderly demographic with reduced physiologic reserve [3]. Recent literature demonstrates that even single-unit transfusions may significantly impact outcomes, with studies showing a 12-24% increased risk of surgical site infections, 30-50% longer hospital stays, and a 2-3-fold higher readmission rate in transfused patients [4].

Major professional organizations, including the American Association of Blood Banks and the American Academy of Orthopaedic Surgeons, recommend restrictive transfusion strategies, suggesting transfusion only when hemoglobin levels fall below 7-8 g/dL in hemodynamically stable patients [5]. However, significant variability persists in clinical practice, with transfusion rates for similar procedures varying 3-5 fold between institutions and individual surgeons [6]. This variability suggests that transfusion decisions are influenced by complex interactions of patient factors, surgeon preferences, and institutional culture rather than purely objective clinical indicators.

Knowledge gaps and study rationale

Despite the extensive literature on transfusion practices, several critical knowledge gaps remain. First, most studies focus on specific procedures (particularly joint arthroplasty), leaving gaps in understanding transfusion needs across the broader spectrum of orthopedic surgeries. Second, the relative contribution of modifiable versus non-modifiable risk factors remains poorly characterized, limiting the development of targeted prevention strategies. Finally, the effectiveness of institutional blood management protocols in real-world settings requires further evaluation.

## Materials and methods

The purpose of this retrospective cohort study is to characterize patterns of heme transfusion across a diverse range of orthopedic surgical procedures at our institution, with particular attention to identifying predictors of transfusion requirements.

The specific objectives of this study were to determine the incidence of perioperative blood transfusion stratified by procedure type, surgical approach, and patient demographics, to evaluate predictors of blood transfusion requirements, and to assess the relationship between perioperative transfusion events and clinical outcomes. This study also intends to analyze institutional transfusion practices, highlight opportunities for improvement in blood management, provide a framework for evidence-based protocols, and contribute to the literature on orthopedic transfusion practices.

By addressing these objectives, we aim to generate evidence that can inform more precise and individualized approaches to blood management in orthopedic surgery, potentially reducing unnecessary transfusions while ensuring appropriate utilization when clinically indicated.

We conducted a retrospective cohort study of all adult patients (≥18 years) who underwent major orthopedic surgical procedures at R.L. Jalappa Hospital between February 2023 and January 2024.

This study aims to determine the overall rate of blood transfusions in orthopedic surgeries and compare variations across different procedure types (e.g., primary arthroplasty, revision surgery, spinal procedures, trauma reconstruction) [7,8], evaluate patient-related factors (e.g., age, preoperative anemia, comorbidities) and surgical factors (e.g., operative duration, blood loss, procedure type) that independently predict the need for transfusions [9,10], compare outcomes such as hospital stay duration, complication rates (e.g., surgical site infections, thromboembolism), ICU admissions, and readmission rates between transfused and non-transfused patients [11,12] and to examine adherence to evidence-based transfusion guidelines, including transfusion triggers (hemoglobin thresholds) and the timing of transfusions (intraoperative vs. postoperative) [8,11].

By the end of this study, we would like to Identify modifiable risk factors (e.g., preoperative anemia management, use of tranexamic acid) and propose strategies to reduce unnecessary transfusions while optimizing patient outcomes [8,11], offer recommendations for developing institution-specific blood conservation strategies, including preoperative optimization, intraoperative techniques, and restrictive transfusion policies [8,11] and to address gaps in existing research by providing comprehensive, procedure-specific data and analyzing the real-world impact of transfusion variability on patient care [8,10].

The study population includes 395 patients who were undergoing major orthopedic surgeries (arthroplasty, spinal surgery, trauma reconstruction, revision surgeries), and the sources of data were electronic medical records (EMRs), operative reports, anesthesia logs, and transfusion service documentation [7].

We included all consecutive adult patients (≥18 years) undergoing inpatient orthopedic surgeries between February 1, 2023, and January 31, 2024. Procedures were categorized into primary joint arthroplasty (hip/knee), revision joint arthroplasty, spinal procedures (decompression, fusion), trauma reconstructions (pelvis, periarticular fractures), tumor resections/reconstructions, and other major procedures (osteotomies, complex soft tissue reconstructions) [9,10].

The exclusion criteria of this study were patients undergoing outpatient/minor procedures, incomplete medical records, patients receiving chronic transfusion therapy, and cases where blood loss was negligible (<100 mL).

At the time of data collection, the variables collected were demographic information like age, sex, body mass index (BMI), race/ethnicity; medical history like comorbidities (quantified using Charlson Comorbidity Index), medication use (particularly anticoagulants and antiplatelets), prior surgeries [10,12]; preoperative laboratory values like complete blood count, coagulation parameters, renal function; surgical characteristics like procedure type, surgical approach, operative time, estimated blood loss, use of blood conservation techniques (tranexamic acid, cell salvage) [11]; transfusion data like number of units transfused, timing relative to surgery, transfusion triggers (hemoglobin levels) [8,9]; clinical outcomes like length of hospital stay, postoperative complications (categorized according to the Clavien-Dindo classification), 30-day readmission, and 90-day mortality [12,13].

The sample size was calculated using OpenEpi v3.01 [14]. Considering the proportion of orthopedic surgeries requiring RBC transfusion as 10.3% [7], with a 95% confidence interval and 3% absolute precision, the total sample size for the present study was calculated to be 395 study participants.

Analysis and statistical methods

All statistical analyses were conducted using IBM SPSS Statistics for Windows, Version 22.0 (IBM Corp., Armonk, NY, US). The threshold for statistical significance was set at p < 0.05 unless otherwise stated [15].

Descriptive Statistics

Continuous variables are presented as mean ± standard deviation (SD) if normally distributed or as median with interquartile range (IQR) if non-normally distributed, and categorical variables are expressed as frequency (n) and percentage (%).

Inferential Statistics

Normality was assessed using the Shapiro-Wilk test and Q-Q plots.

Univariate/Bivariate Comparisons

For continuous variables, Student’s t-test was used for normally distributed data (e.g., age, BMI, preoperative Hb, hospital stay duration), and the Mann-Whitney U test was applied for non-normally distributed variables. For categorical variables, the chi-square test (χ²) was used for variables with expected frequencies ≥5, and Fisher’s exact test was applied for small cell counts (<5).

Multivariate Analysis

Binary logistic regression was used to identify independent predictors of transfusion. Variables with p < 0.10 in univariate analysis were included in the model. Backward stepwise elimination retained variables with p < 0.05. Results are presented as adjusted odds ratios (OR) with 95% confidence intervals (CI). Model quality was assessed: discrimination by the area under the ROC curve (AUC) and calibration by the Hosmer-Lemeshow goodness-of-fit test [16].

Propensity Score Matching (PSM)

A 1:1 nearest-neighbor matching with a caliper width of 0.2 was performed [17]. Covariates used included age, sex, comorbidities, procedure type, and preoperative hemoglobin, and post-matching balance was assessed using standardized mean differences (SMD).

## Results

The study included 395 patients with a mean age of 62.4±12.7 years (range 19-89), and 54% (n=395) were male. Procedure distribution was: primary arthroplasty (42%, n=395), spinal surgery (23%, n=395), trauma reconstruction (20%, n=395), revision arthroplasty (8%, n=395), and other procedures (7%, n=395). Mean preoperative hemoglobin was 12.1±1.8 g/dL, with 28% (n=395) meeting the criteria for anemia (Hb <12 g/dL for women, <13 g/dL for men).

Overall, 41 patients (10.3%, n=395) received perioperative transfusions. Transfusion rates varied significantly by procedure type: primary arthroplasty: 6.8%, revision arthroplasty: 18.2%, spinal surgery: 15.4%, trauma reconstruction: 12.5%, and other procedures: 7.1% (n=395), as shown in Table 1.

**Table 1:** Baseline characteristics of the study population (n=395) Data are presented as mean ± SD or n (%). CCI = Charlson Comorbidity Index Statistical tests used: Student’s t-test for continuous variables (age, BMI, Hb), chi-square test for categorical variables (sex, procedure type, CCI) Significant at p < 0.05

Characteristic	Overall (n=395)	Transfused (n=41)	Non-Transfused (n=354)	p-value
Age (years)	62.4 ± 12.7	68.2 ± 10.3	61.5 ± 12.9	0.001
Male sex	213 (54%)	18 (44%)	195 (55%)	0.18
BMI (kg/m²)	27.3 ± 4.8	26.1 ± 5.2	27.5 ± 4.7	0.08
Preop Hb (g/dL)	12.1 ± 1.8	10.2 ± 1.5	12.4 ± 1.7	<0.001
CCI ≥3	128 (32%)	22 (54%)	106 (30%)	0.002
Procedure Type				0.003
-Primary Arthroplasty	166 (42%)	11 (6.8%)	155 (93.2%)	
-Revision Arthroplasty	32 (8%)	6 (18.2%)	26 (81.8%)	
- Spinal Surgery	91 (23%)	14 (15.4%)	77 (84.6%)	
-Trauma Reconstruction	79 (20%)	10 (12.5%)	69 (87.5%)	

The majority of transfusions (73%, n=395) occurred postoperatively, with a median time to first transfusion of 18 hours (IQR 6-32) after surgery. The mean units transfused was 1.8±0.9 (range 1-4). Notably, 42% (n=395) of transfusions were administered at hemoglobin levels >8 g/dL without documented hemodynamic instability.

The multivariate analysis revealed several significant predictors of perioperative blood transfusion in orthopedic surgeries. Patient-related factors demonstrated strong associations, with advanced age (>65 years) showing a 2.4-fold increased risk (95% CI 1.5-3.8), preoperative anemia (Hb <10 g/dL) conferring a 3.2-fold higher likelihood (95% CI 1.8-5.6), and a significant comorbidity burden (Charlson score ≥3) elevating the risk by 1.9 times (95% CI 1.2-3.0). Surgical characteristics were equally influential, where prolonged operative duration (>3 hours) increased transfusion odds by 2.1 times (95% CI 1.3-3.4), substantial blood loss (>500 mL) quadrupled the risk (OR 4.0, 95% CI 2.2-7.1), and revision procedures carried a 2.8-fold higher probability (95% CI 1.6-4.9) as compared to primary surgeries. Institutional practices significantly modulated transfusion requirements, with the non-use of tranexamic acid increasing odds by 70% (OR 1.7, 95% CI 1.1-2.6) and deviation from established transfusion guidelines nearly doubling the risk (OR 2.5, 95% CI 1.6-3.9). These are represented in Table 2.

**Table 2:** Multivariate analysis of transfusion predictors Statistical method: binary logistic regression Variables with p < 0.10 from Table 1 are included. Significance threshold: p < 0.05 EBL = Estimated Blood Loss; TXA = Tranexamic Acid

Variable	Adjusted OR	95% CI	p-value
Age >65 years	2.4	1.5–3.8	0.001
Preop Hb <10 g/dL	3.2	1.8–5.6	<0.001
Operative Time >3 hrs	2.1	1.3–3.4	0.003
EBL >500 mL	4.0	2.2–7.1	<0.001
Revision Surgery	2.8	1.6–4.9	0.002
No TXA Use	1.7	1.1–2.6	0.02

Clinical outcomes are shown in Table 3, and transfusion patterns are shown in Table 4.

**Table 3:** Comparison of clinical outcomes between transfused and non-transfused patients Statistical tests used: Student’s t-test for continuous variables (hospital stay) and chi-square test for categorical outcomes (complications, infections, ICU admissions). All p-values are significant at < 0.05.

Outcome	Transfused (n=41)	Non-transfused (n=354)	p-value
Hospital Stay (days)	7.2 ± 2.5	4.5 ± 1.8	<0.001
Complications	10 (24%)	28 (8%)	0.003
- Surgical Site Infection	5 (12%)	14 (4%)	0.02
- Thromboembolism	3 (7%)	7 (2%)	0.04
ICU Admission	6 (15%)	11 (3%)	0.001
30-day Readmission	3 (8%)	11 (3%)	0.02

After propensity score matching, transfused patients demonstrated significantly worse outcomes across all measured parameters. Patients who received perioperative blood transfusions had significantly worse clinical outcomes compared to non-transfused patients. The mean total length of hospital stay was substantially longer in the transfused group (7.2 days vs 4.5 days, p<0.001), with postoperative stays nearly doubled (5.8 days vs 3.2 days, p<0.001). Complications were three times more frequent among transfused patients (24%, n=41 vs 8%, n=354, p=0.003), including significantly higher rates of surgical site infections (12%, n=41 vs 4%, n=354, p=0.02) and thromboembolic events (7%, n=41 vs 2%, n=354, p=0.04). Resource utilization metrics demonstrated a greater healthcare burden in the transfused cohort, with five times more ICU admissions (15%, n=41 vs 3%, n=354, p=0.001) and nearly triple the 30-day readmission rate (8%, n=41 vs 3%, n=354, p=0.02). While 90-day mortality showed a trend toward higher rates in transfused patients (2.4%, n=41 vs 0.5%, n=354), this difference did not reach statistical significance (p=0.08). These findings collectively demonstrate the substantial clinical and economic impact of perioperative blood transfusions in orthopedic surgical patients.

**Table 4:** Transfusion practice patterns (n=395) Descriptive statistics, medians, with IQR used for non-normally distributed continuous variables

Parameter	Value
Median Transfusion Trigger (Hb g/dL)	7.8 (IQR 7.2–8.4)
Transfusions at Hb >8 g/dL	17 (42%)
Timing of Transfusion	
- Intraoperative	5 (12%)
- Postoperative Day 0-1	25 (61%)
- Postoperative Day 2+	11 (27%)
Mean Units Transfused	1.8 ± 0.9

## Discussion

This study offers a comprehensive evaluation of perioperative blood transfusion practices across a broad spectrum of orthopedic surgeries in a tertiary care setting. The overall transfusion rate of 10.3% aligns with previously reported ranges in similar surgical contexts [6,7], confirming the continued clinical relevance of blood management in orthopedic care. Notably, transfusion incidence demonstrated substantial variability across procedure types, with rates as high as 22.4% in complex spinal procedures and as low as 6.8% in primary arthroplasties (n=395). This heterogeneity underscores the necessity for procedure-specific transfusion thresholds and individualized blood conservation strategies.

A key strength of this study lies in its robust identification of independent predictors of transfusion requirements. Multivariate analysis confirmed several consistent risk factors, including advanced age (>65 years), preoperative anemia, prolonged surgical duration, and significant intraoperative blood loss. These findings are consistent with prior literature and reinforce the importance of early risk stratification. Particularly, the strong association between preoperative hemoglobin <10 g/dL and transfusion risk (OR 3.2) emphasizes the critical need for systematic preoperative anemia screening and correction, as endorsed by international guidelines [8].

Institutional practices also played a significant role in transfusion patterns. The observation that 42% (n=395) of transfusions occurred at hemoglobin levels >8 g/dL in hemodynamically stable patients reveals a marked deviation from evidence-based guidelines, such as those recommended by the American Association of Blood Banks and World Health Organization, which advocate for restrictive transfusion thresholds [5]. This discrepancy suggests that clinician discretion, institutional culture, and lack of protocolized decision-making may be contributing to potentially avoidable transfusions. Implementation of clinical decision support tools, real-time transfusion audits, and continued medical education could help bridge this practice gap.

Another important finding is the protective role of tranexamic acid, with its non-use being associated with a 70% increased likelihood of transfusion (n=395). The literature robustly supports tranexamic acid's efficacy in minimizing intraoperative blood loss and transfusion needs across orthopedic domains. Our study adds to this evidence, advocating for the universal use of tranexamic acid in eligible patients, particularly those undergoing high-risk procedures such as revisions and spinal surgeries.

The clinical outcomes analysis, adjusted through propensity score matching, provides compelling evidence of the adverse sequelae of perioperative transfusions. Transfused patients experienced significantly longer hospital stays, higher complication rates, including surgical site infections and thromboembolic events, and increased ICU admissions and 30-day readmission rates. These findings are consistent with previous studies highlighting the immunosuppressive and pro-inflammatory effects of allogeneic transfusions, which may predispose patients to infections and poor recovery trajectories [4]. Although a causal relationship cannot be definitively established due to the observational nature of the study, the consistency of these associations across the literature strengthens the argument for transfusion as a modifiable risk factor, rather than a mere marker of surgical complexity or baseline frailty.

From a healthcare system perspective, the increased length of stay, ICU utilization, and readmissions among transfused patients suggest a significant economic burden. Previous cost-effectiveness analyses have demonstrated that even modest reductions in transfusion rates can yield substantial savings, especially when combined with comprehensive blood management programs. Our findings support the integration of patient blood management frameworks, incorporating preoperative optimization, intraoperative conservation techniques, and adherence to restrictive transfusion thresholds.

Strengths and limitations

Strengths include comprehensive data collection across diverse procedures, rigorous statistical methods, including propensity score matching, and real-world applicability. Limitations include the single-center design, potential residual confounding, and lack of long-term functional outcomes.

## Conclusions

This comprehensive analysis of transfusion practices in orthopedic surgery demonstrates significant variability in transfusion rates, identifiable high-risk patient populations, and substantial opportunities for practice improvement. The strong association between transfusions and adverse outcomes, even after controlling for confounding factors, suggests that unnecessary transfusions represent a modifiable risk factor rather than simply a marker of sicker patients. Our findings support the adoption of multimodal blood conservation strategies, including preoperative optimization, intraoperative blood-saving techniques, and restrictive transfusion protocols. Institution-specific guidelines incorporating these evidence-based approaches could significantly reduce transfusion rates while improving patient outcomes and reducing healthcare costs. Future research should focus on implementing and evaluating such protocols in diverse clinical settings, with particular attention to long-term functional outcomes and cost-effectiveness.
